# Microbiome and metabolomics analyses of the effect of heat-sensitive moxibustion on allergic rhinitis in rats

**DOI:** 10.3389/fimmu.2025.1656060

**Published:** 2025-10-31

**Authors:** Jun Xiong, Jianheng Li, Haiyan Xu, Yicheng Li, Lunbin Lu, Shuang Yang

**Affiliations:** ^1^ Department of Traditional Chinese Medicine, The Second Affiliated Hospital of Nanchang University, Nanchang, China; ^2^ Graduate School, Jiangxi University of Chinese Medicine, Nanchang, China; ^3^ Clinic and Center for Public Health Education & Health Services, Jiangxi University of Chinese Medicine, Nanchang, China; ^4^ Second Clinical Medical College, Nanchang University, Nanchang, China

**Keywords:** allergic rhinitis, biomarkers, metabolomics, microbiome, multiomics

## Abstract

**Background:**

The original concept of acupoint sensitization theory was put forward in Huangdi Neijing, which believed that acupoints, as the reflecting parts of the body surface, are personalized, changeable, and sensitive. Heat-sensitive moxibustion has a good therapeutic effect on allergic rhinitis, but the mechanism is still unclear. Notably, acupoint sensitization in allergic rhinitis (AR) rats was accompanied by a distal thermal effect, with an increase in tail temperature (TTI) after 40 min of moxibustion.

**Objective:**

The objective was to utilize multi-omics techniques and correlation analysis to explore the unique mechanisms of heat-sensitive moxibustion in intervening in AR compared with traditional moxibustion from the perspectives of gut microbiota and metabolites.

**Methods:**

Thirty-six Sprague–Dawley (SD) rats were randomly divided into two groups: the ovalbumin (OVA) group (n = 27) and the control group (Con) (n = 9). The rat model of AR induced by standardized OVA was established through intranasal infusion after intraperitoneal OVA injection. Through behavioral scoring, nasal symptoms were evaluated, including nasal scratching, runny nose, and sneezing, to ensure the success of the modeling. The OVA group was randomly divided into the moxibustion group (n = 17) and the AR group (n = 8). Then, through suspended moxibustion for 40 min, they were divided into TTI, namely, the heat-sensitive moxibustion group (HM) (n = 8) and the non-TTI group (OM) (n = 8), and one subject was excluded. The levels of serum IL-4 and IgE were quantified by enzyme-linked immunosorbent assay (ELISA), and the histological characteristics of nasal tissues were evaluated by hematoxylin and eosin (H&E) staining to determine the reliability of the AR rat model and the effectiveness of thermal sensitization. The V3 and V4 regions of the 16S ribosomal DNA (rDNA) gene were analyzed from rat feces using 16S rDNA sequencing technology. In addition, non-targeted metabolomics was used to identify the differential metabolites in rat urine. Finally, through the comparison and correlation analysis of different bacterial microbiota and metabolites, we aimed to clarify the unique material basis of heat-sensitive moxibustion in the context of AR.

**Results:**

After the OVA modeling was completed, through behavioral score evaluation, we found that there were differences between the OVA group and the control group. After the intervention treatment, it was found that the levels of IgE and IL-4 in the AR group were significantly higher than those in the control group. Staining showed that moxibustion relieved nasal symptoms, and the thermal sensitization effect was satisfactory. We noticed that significant changes occurred in the flora under heat-sensitive moxibustion treatment. We investigated the mechanism of HM in treating AR using an integrated 16S rRNA sequencing technology and untargeted metabolomics. Our results showed that HM treatment ameliorated AR in rats. The high-throughput sequencing results indicate that HM significantly increased the relative abundance of species, such as Patescibacteria, Saccharimonadaceae, UCG-010, *Butyrivibrio*, *Turicibacter*, *Lactobacillus murinus*, and *Adlercreutzia*, while decreasing the relative abundance of Prevotellaceae. This shift in microbial composition is conducive to improving the gut microbiota of AR rats. Untargeted metabolomics results showed that HM treatment regulated the metabolites such as 1-methylhistidine, xi-3-hydroxy-5-phenylpentanoic acid *O*-beta-d-glucopyranoside, cladosporin, cuminaldehyde, daidzein, Pe(18:0/15:0), *N*-nervonoyl asparagine, edulitine, *N*-arachidonoyl glycine, 9alpha-(3-methyl-2*E*-pentenoyloxy)-4*S*-hydroxy-10(14)-oplopen-3-one, quisqualic acid, ethyl glucuronide, zileuton *O*-glucuronide, trichloroethanol glucuronide, Asp Leu Ser Glu, quinolinic acid, and norvaline. We finally identified six crossing pathways by pin-to-pair comparison of three groups: glutamatergic synapse, dopaminergic synapse, Kaposi sarcoma-associated herpesvirus infection, cocaine addiction, melanin production, alcoholism, and histidine metabolism. Subsequently, we focused on studying the histidine metabolism. To clarify the changes in the activity of this pathway, we measured the histamine content using an enzyme-linked immunosorbent assay. Compared with the OM group, we found that HM had a trend toward superior efficacy in reducing tissue histamine compared to OM. The histamine content in the HM group was significantly lower than that in the OM group. This finding suggests that HM is more effective in reducing histamine, and its effect may be related to a more efficient regulation of the histidine metabolic pathway.

**Conclusions:**

This study demonstrates that heat-sensitive moxibustion alleviates allergic rhinitis through a multi-targeted mechanism involving both the modulation of specific gut microbiota (notably *L. murinus*, Patescibacteria, *Butyrivibrio*, and *Turicibacter*)—which is closely associated with alterations in key metabolites (cuminaldehyde and 1-methylhistidine)—and the regulation of histidine metabolism. To our knowledge, this represents the first investigation to establish comprehensive correlations between gut microbiota and urinary metabolomics profiles in an AR model. Our findings confirm the therapeutic role of heat-sensitive moxibustion in AR recovery and provide mechanistic insights supporting its clinical application, thereby proposing a novel strategic approach for AR treatment.

## 1 Introduction

Allergic rhinitis (AR) is a type I allergic disease mediated by IgE, currently affecting up to 400 million people worldwide, and its incidence is increasing year by year (1). According to the survey, the prevalence of AR in first-tier cities in China shows a significant upward trend. The main manifestations of nasal symptoms include nasal itching, sneezing, runny nose, and nasal congestion; long-term development may be combined with asthma, accompanied by cough, wheezing, shortness of breath, chest tightness, and other respiratory system lesions (2). It will not only lead to anxiety, depression, and other mental stress but also cause a decline in labor efficiency (3). According to the statistics of EU countries, AR can cause economic losses of 30 billion to 50 billion euros per year, which not only endangers physical and mental health but also affects economic and social development (4).

The modern medical treatment of AR is mainly drug therapy and immunotherapy. Medications such as antihistamines and corticosteroids have many side effects (5). In addition, allergen-specific immunotherapy is the only specific treatment plan, but it also faces problems such as efficacy, safety, and long-term continuous treatment of patient compliance. Therefore, it is urgent to seek a safer and more effective AR treatment in the long term. In this regard, we turned our interest to heat-sensitive moxibustion (6).

Moxibustion, a traditional Chinese medicine (TCM) therapy, is widely used in China to treat chronic conditions such as allergic rhinitis. Suspended moxibustion, an indirect form of this therapy, involves holding burning moxa several centimeters above the skin without direct contact. TCM theory posits that acupoints exist in two states: active (sensitive) and dormant (resting). During illness, surface acupoints become stimulated and sensitized. These areas, termed “heat-sensitive points”, readily respond to thermal stimulation. Stimulating these points with moxibustion often triggers a detectable thermal response in distant body regions, observable through physical measurements. This distant thermal response significantly enhances the therapeutic efficacy of moxibustion (7). The *Huangdi Neijing* (*Yellow Emperor’s Inner Canon*) articulates the foundational theory behind this sensitization phenomenon. Acupoints are defined by their dynamic states, which manifest as specific skin areas altered during disease progression. Sensitized states are common in such cases, where sensitized acupoints exhibit a “minor stimulus, amplified response” effect—a unique reactivity to external stimuli. In this context, the “minor stimulus” refers to moxibustion applied to sensitized acupoints. During suspended moxibustion therapy, patients first report distant heat sensations approximately 15 min into treatment, followed by a rapid increase in intensity that persists until the session concludes (8).

Recent advancements in thermal-sensitive suspended moxibustion have significantly enhanced the therapeutic effectiveness of traditional moxibustion practices. Controlled clinical studies have demonstrated that thermal-sensitive moxibustion exhibits superior clinical efficacy in managing AR compared to conventional moxibustion approaches (9, 10).

The gut–microbiota–metabolism axis has emerged as a prominent research focus in contemporary medicine, with robust evidence establishing significant associations between microbial dysbiosis and AR pathogenesis (11, 12).

Metabolomics analyzes biological samples—including blood, urine, and stool—to identify small molecules linked to health or disease states. It detects variations in these molecules, which can aid in diagnosing diseases or screening potential therapies. Metabolomics profiles reflect the cumulative effects of environmental, dietary, or microbial exposures (13). Because metabolites directly reflect microbial activity, they correlate strongly with microbiome composition and function. Combining microbiome and metabolomics data can uncover novel insights into disease mechanisms and therapeutic targets (14).

The gut microbiome, a complex microbial community, plays a vital role in regulating immune responses and inflammation (15). Recent studies have indicated that AR alters gut microbiome composition and significantly contributes to disease development. Investigating interactions between the microbiome and metabolome offers novel insights into AR mechanisms, providing a foundation for innovative therapeutic strategies. Modulating gut microbiota may lead to new treatment approaches for AR. This study supports the clinical use of heat-sensitive moxibustion for AR and demonstrates how integrating metabolomics and microbiome research can advance therapies for related conditions.

## 2 Materials and methods

### 2.1 Materials

Heat-sensitive moxibustion was purchased from the Affiliated Hospital of Jiangxi University of Traditional Chinese Medicine (Nanchang, China); ovalbumin (OVA; American Sigma, St. Louis, Missouri) and aluminum hydroxide (Tianjin Damao, Tianjin, China) were used. Plasma was used to determine IL-4 and IgE levels using an enzyme-linked immunosorbent assay (ELISA) kit (Shenzhen Xinbosheng); nasal mucosa was used to determine histamine levels using an ELISA kit (Jiangsu Meimian, Jiangsu, China).

### 2.2 Animals

Thirty-six male Sprague–Dawley (SD) rats (5 weeks old, 151–175 g) were purchased from Hunan Sheng Hua Laboratory Animal Co., Ltd. (Hunan Province, China). The rats were housed at the Experimental Animal Center of Jiangxi University of Chinese Medicine under controlled conditions: 12-hour light/dark cycles, 22 °C–26 °C room temperature, 50%–65% humidity, and adequate ventilation. All procedures were approved by Jiangxi Medical University’s Institutional Animal Care and Use Committee (IACUC; Approval No. JZLLSC20240539) and conducted in accordance with institutional guidelines.

### 2.3 Establishment of rat AR model

A total of 36 rats were randomly divided into two groups: blank group (n = 9) and OVA group (n = 27) (16). According to the previous literature, an AR model was established (17). During the sensitization phase (days 1–14), the control group received standard feed without interventions. The model group received intraperitoneal injections of sensitizing solution [0.3 mg ovalbumin (OVA) + 30 mg aluminum hydroxide in 1 mL saline] every other day for seven doses. On day 15, the model group was further sensitized with daily intranasal administration of 25 μg 10% OVA for seven consecutive days. Of 27 rats subjected to OVA sensitization, one died due to improper injection and one from nasal hemorrhage during modeling. Post-treatment symptom scores (sneezing, nasal scratching, and discharge) were assessed by blinded observers. A total score >5 indicated successful AR modeling. Rats were randomized into a model group (n = 8) and a moxibustion treatment group (n = 17).

### 2.4 Heat-sensitive moxibustion

To facilitate moxibustion, rats were gently restrained in a ventilated cage that allowed natural posture while minimizing movement. The room temperature was kept at 25.2 °C during the whole experiment. Lung acupuncture point (BL13), which is very important for AR, is the commonly used acupuncture point for AR clinical treatment and rat experiments (18, 19). Moxibustion was applied to depilated skin at a height of 3 cm using animal-specific moxa sticks (12-cm length, 0.6-cm diameter; produced by the Affiliated Hospital of Jiangxi University of Chinese Medicine). Both the heat-sensitive and standard moxibustion groups received daily 40-min sessions at the Feishu acupoint (BL13) for three consecutive weeks. During this period, the moxa stick was fixed onto the moxibustion stand.

### 2.5 Tail temperature measurement

**Figure 1:**
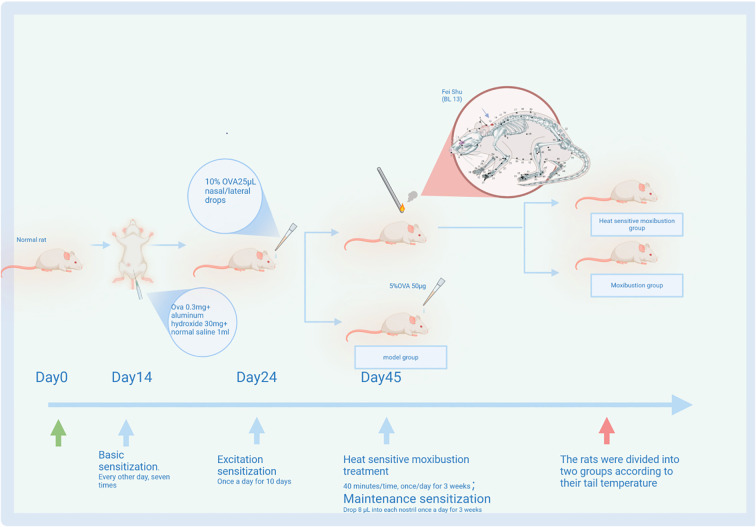
Establishment of allergic rhinitis (AR) model mice and heat-sensitive moxibustion treatment plan.

The mid-tail temperature of rats was measured using a digital thermometer (Shanghai Medical Equipment Factory, Shanghai, China). Measurements were taken in a quiet environment with controlled room temperature (25 °C ± 2 °C). Before the experiment, rats were acclimated in their cages for 30 min. During the treatment phase, all three groups received ongoing sensitization with 5% OVA solution every 5 days. After heat-sensitive moxibustion treatment, one rat was excluded because its tail temperature response was indeterminate and deemed to be at the threshold for defining heat-sensitive responsiveness, ensuring the purity of the HM group phenotype. Based on tail temperature changes, the moxibustion group was subdivided into two subgroups—a non-elevated subgroup (≤1 °C, OM group, n = 8) and an elevated subgroup (>1 °C, HM group, n = 8 (16))—determined by average measurements over 7 days. The experimental protocol for OVA-induced AR and heat-sensitive moxibustion is summarized in **Figure 1**. After 40 min of treatment, there were some rats exhibiting an increase in tail temperature (TTI) (more than 2°C on average) in the moxibustion group (**Table 1**). The occurrence rate of TTI is eight out of 17 (47.05%). Furthermore, the temperature began to rise within approximately 10 to 15 min and then decreased within 30 to 40 min, lasting for approximately 20 to 25 min each time. There was no change in the tail temperature of the model group without treatment.

**Table 1:** The change in the tail temperature of mice under moxibustion treatment.

Subgroups	Tail temperature increase			
	≥1 °C HM		≤1 °C OM	
	2.82 ± 0.22	2.56 ± 0.34	0.67 ± 0.21	0.64 ± 0.37
	2.45 ± 0.21	2.48 ± 0.26	0.75 ± 0.19	0.83 ± 0.21
	2.65 ± 0.21	2.64 ± 0.25	0.82 ± 0.23	0.81 ± 0.24
	2.75 ± 0.30	2.44 ± 0.26	0.85 ± 0.16	0.78 ± 0.32^*^
Total	8		8	

### 2.6 Symptom score

Symptom scores after the final treatment were assessed by observation of sneezing, nose scratching, and runny nose in rats and were unknown to the observer. A total score of more than 5 indicates that the AR model is successfully established, and the rats fluctuating around 1 °C were not included in the observation objects. After treatment in the moxibustion group, one rat was excluded because it could not be determined whether its tail temperatures were heat-sensitized.

### 2.7 Sample collection

After overnight fasting, 12-hour urine samples were collected from individually housed rats in metabolic cages (8:00 PM to 8:00 AM). Urine was centrifuged at 4 °C and 800 × *g* for 10 min to remove debris, and the supernatant was retained. After centrifugation at 12,000 ×*g* and 4 °C for 10 min, the clarified middle layer was aspirated, aliquoted into 1.5-mL tubes (1 mL per aliquot), and stored at −80 °C. Prior to dissection, rats were restrained by securing the neck, spine, and tail. Fecal samples were collected via stress-induced defecation onto sterile paper (replaced for each rat) and transferred to sterile microcentrifuge tubes using disinfected toothpicks. Fecal pellets (one to two per rat) were placed in sterile tubes, flash-frozen in liquid nitrogen for 5 min, and stored at −80 °C. Rats were euthanized via intraperitoneal injection of xylazine (200 μg/rat) and ketamine (2 mg/rat). Blood was collected from the brachial artery, centrifuged to isolate serum, and stored at −80 °C for ELISA analysis. The nasal septa of the rats were collected and placed in the fixative solution.

### 2.8 Enzyme-linked immunosorbent assay

Blood was collected in a tube containing Ethylenediaminetetraacetic acid (EDTA) and centrifuged (3,500 × *g*, 4 °C, 10 min) to separate the blood. IL-4 and IgE levels were measured in plasma using an ELISA kit.

### 2.9 Hematoxylin and eosin and immunohistochemistry staining

Mouse nasal septa were fixed in 4% paraformaldehyde, followed by decalcification in EDTA solution for 9 days, and embedded in paraffin. Tissue sections (5-μm thickness) were prepared and stained with hematoxylin and eosin (H&E).

### 2.10 16S rDNA amplicon sequencing

Total microbial genomic DNA was extracted from rat dropping samples using the FastPure Stool DNA Isolation Kit (MJYH, Shanghai, China) according to the manufacturer’s instructions. The quality and concentration of DNA were determined by 1.0% agarose gel electrophoresis and a NanoDrop2000 spectrophotometer (Thermo Fisher Scientific, Waltham, Massachusetts, USA) and kept at −80 °C prior to further use. The hypervariable regions V3–V4 of the bacterial 16S rRNA gene were amplified with primer pairs 338F (ACTCCTACGGGAGGCAGCAG) and 806R (GGACTACHVGGGTWTCTAAT) using the T100 Thermal Cycler PCR thermocycler (BIO-RAD, Hercules, California, USA). The PCR mixture included 4 μL 5 × Fast Pfu buffer, 2 μL 2.5 mM dNTPs, 0.8 μL each primer (5 μM), 0.4 μL Fast Pfu polymerase, 10 ng of template DNA, and ddH_2_O to a final volume of 20 µL. PCR amplification cycling conditions were as follows: initial denaturation at 95 °C for 3 min, followed by 27 cycles of denaturing at 95 °C for 30 s, annealing at 55 °C for 30 s, extension at 72 °C for 45 s, and a single extension at 72 °C for 10 min, and ended at 4 °C. The PCR product was extracted from 2% agarose gel and purified using the PCR Clean-Up Kit (YuHua, Shanghai, China) according to the manufacturer’s instructions and quantified using Qubit 4.0 (Thermo Fisher Scientific, USA).

### 2.11 Metabolomic analysis

Urine sample (200 μL) was transferred to a 1.5-mL centrifuge tube, and 400 μL of extract (acetonitrile:methanol = 1:1) was added. After vortex mixing for 30 s, cold ultrasonic extraction was performed for 30 min (5 °C, 40 kHz), and the sample was allowed to stand at −20 °C for 30 min and then at 4 °C. After centrifugation at 13,000 × *g* for 15 min, the supernatant was removed and dried using nitrogen, and 100 µL of compound solution (acetonitrile:water = 1:1) was reconstituted. Low-temperature ultrasonic extraction for 5 min (5 °C, 40 kHz) and centrifugation at 13,000 × *g* for 5 min were performed to remove the supernatant, which was transferred to a vial. In addition, 20 µL of the supernatant from each sample was used as a quality control sample to investigate the repeatability of the whole analysis process.

The instrument platform used for this Liquid Chromatography-Mass Spectrometry (LC–MS) analysis was Thermo Scientific ultra-high performance liquid chromatography–tandem Fourier transform mass spectrometry UHPLC-Q Exactive HF-X system. Chromatographic conditions were as follows: 2-μL samples were separated using an HSS T3 column (100 mm × 2.1 mm i.d., 1.8 µm) and then detected using mass spectrometry. Mobile phase A was 95% water + 5% acetonitrile (containing 0.1% formic acid), and mobile phase B was 47.5% acetonitrile + 47.5% isopropyl alcohol + 5% water (containing 0.1% formic acid). Separation gradient was performed as follows: at 0–3.5 min, mobile phase B increased from 0% to 24.5%, and the flow rate was 0.40 mL/min. At 3.5–5 min, mobile phase B increased from 24.5% to 65%, and the flow rate was 0.4 mL/min. At 5–5.5 min, mobile phase B increased from 65% to 100%, and the flow rate was 0.4 mL/min. At 5.5–7.4 min, mobile phase B was maintained at 100%, and the flow rate increased from 0.4 to 0.6 mL/min. At 7.4–7.6 min, mobile phase B decreased from 100% to 51.5%, and the flow rate was 0.6 mL/min. At 7.6–7.8 min, the mobile phase B decreased from 51.5% to 0%, and the flow rate decreased from 0.6 to 0.5 mL/min. At 7.8–9 min, the mobile phase B was maintained at 0%, and the flow rate decreased from 0.5 to 0.4 mL/min. At 9–10 min, 0% mobile phase B was maintained, and the flow rate was 0.4 mL/min. The column temperature was 40 °C.

Mass spectrum conditions were as follows: the sample quality spectrum signal was collected in positive and negative ion scanning modes, and the mass scanning range was 70–1,050 m/z. The flow rate of sheath gas was 50 psi, the flow rate of auxiliary gas was 13 psi, the heating temperature of auxiliary gas was 425 °C, the positive mode ion spray voltage was set to 3,500 V, the negative mode ion spray voltage was set to −3,500 V, the ion transport tube temperature was 325 °C, and the normalized collision energy was 20–40–60 V cyclic collision energy. The primary mass spectral resolution was 60,000, the secondary mass spectral resolution was 7,500, and the data were collected using Data-Dependent Acquisition (DDA) mode.

### 2.12 Statistical analysis

All data analyses were performed on the Megge BioCloud Platform (https://cloud.majorbio.com). The details were as follows: the mothur (20) software (http://www.mothur.org/wiki/Calculators) was used to calculate alpha diversity, which was measured using Chao 1 and Shannon index. Alpha diversity was analyzed using the Wilcoxon rank sum test. Principal coordinates analysis (PCoA) (primary coordinates) was used in combination with the base-based Bray–Curtis distance algorithm, and it was combined with the Permutational Multivariate ANalysis Of Variance (PERMANOVA) non-parametric test to determine whether the difference in microbial community structure between sample groups was significant. Linear discriminant analysis (LDA) effect size (LEfSe) analysis (21) (http://huttenhower.sph.harvard.edu/LEfSe (LDA > 2, p < 0.05) was used to identify bacterial taxa that significantly varied in phylum to genus level abundance between different groups. Network plot analysis (22) was performed to pick species based on Spearman’s correlation |r| > 0.6, p < 0.05.

After computations were completed, LC–MS raw data were imported into the metabolomics processing software Progenesis QI (Waters Corporation, Milford, MA, USA) for baseline filtering, peak identification, integration, retention time correction, and peak alignment. Finally, a data matrix of retention time, mass–charge ratio, and peak intensity was made. The MS and Tandem Mass Spectrometry (MSMS) mass spectrum data were also matched with those in the metabolomics public databases HMDB (http://www.hmdb.ca/) and Metlin (https://metlin.scripps.edu/) and the Meiji self-built library to obtain the metabolite information. After searching, the matrix data were uploaded to Meiji Biological Cloud Platform (https://cloud.majorbio.com) for data analysis. First, for data preprocessing, a data matrix with 80% rules was used to remove the missing value, namely, to keep at least a set of non-zero values of variables, and then the vacancy value (the smallest value in the original matrix) was filled. To reduce the error of the sample preparation and instrument instability, the sample mass spectrum peak response intensity was normalized using the sum normalization method to obtain the normalized data matrix. Variables with relative standard deviation (RSD) >30% were also removed, and log10 logarithm was used to obtain the final data matrix for subsequent analyses.

Differential analysis was performed on the preprocessed matrix files. The R software package ropls (Version 1.6.2) was used to perform principal component analysis (PCA) and orthogonal partial least squares discriminant analysis (OPLS-DA), and a seven-cycle interaction validation was used to evaluate the stability of the model. Furthermore, a Student’s t-test was performed.

Differences in metabolites were distinguished using the KEGG database (https://www.kegg.jp/kegg/pathway.html) on the metabolic pathways of annotation, as well as the differences in metabolites involved in pathways. The Python software package scipy.stats was used for pathway enrichment analysis, and the biological pathways most relevant to experimental treatment were obtained using Fisher’s exact test.

## 3 Results

### 3.1 Heat-sensitive moxibustion alleviates itching, sneezing, and pathological symptoms in OVA-induced AR mice

The behavioral scores of the rats were recorded within 30 min after modeling. The behavioral scores were divided into three symptoms: nose scratching, sneezing, and runny nose. The scores were assigned according to the behavioral score table.

**Figure 2:**
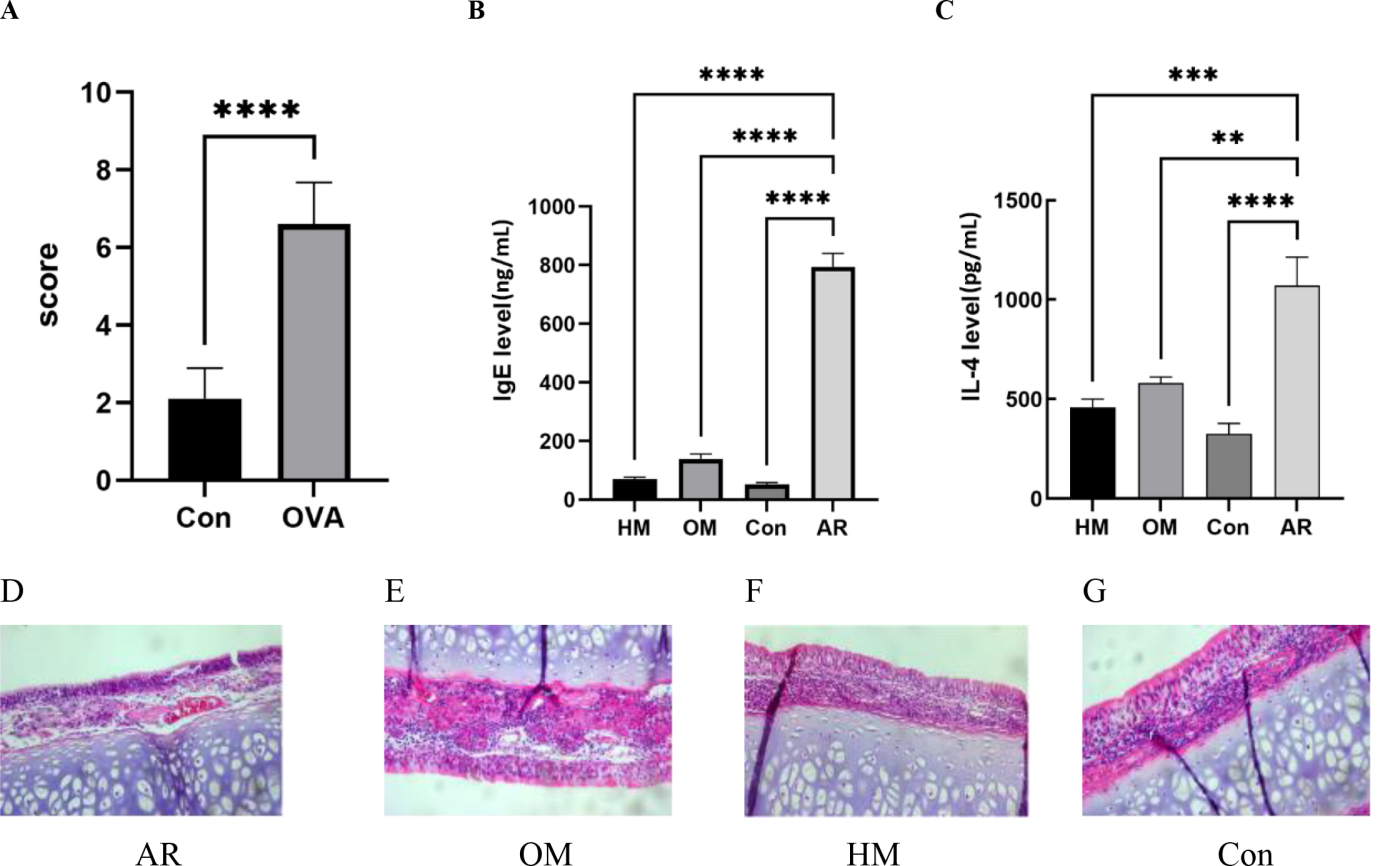
A reliable allergic rhinitis (AR) mouse model was established. **(A)** Behavioral score. Control group (Con) group (n = 27) vs. AR group (n = 9). Heat-sensitive moxibustion (HM) alleviated **(B)** IgE and **(C)** IL-4 in serum (n = 8). **(D–G)** Pathological symptoms. **p < 0.01; ***p < 0.001; ****p < 0.0001.

The rat model of AR induced by OVA exhibited clinical symptoms similar to those of AR, such as sneezing, runny nose, and nasal itching. OVA stimulation significantly increased the incidence of sneezing and friction in rats (p < 0.01), and the modeling was significantly successful (**Figure 2A**). In the OVA group, epithelial cells were disordered, and submucosal eosinophils infiltrated. In the moxibustion group, inflammatory cell infiltration was observed in the mucous lamina propria, epithelial cell arrangement was disordered to a certain extent, and submucosal eosinophils infiltrated. The structure of the heat-sensitive moxibustion group was clear, the mucosal surface cilia were dense, the lamina propria had slight capillary dilatation, and a small amount of eosinophils accumulated, which was significantly reduced compared with that in the model group. The above indicates that heat-sensitive moxibustion is more effective than ordinary moxibustion in reducing nasal symptoms (**Figures 2D–G**).

### 3.2 Heat-sensitive moxibustion alleviates OVA-induced excessive secretion of inflammatory factors

We measured serum IgE and IL-4 levels in different groups of mice. Serum IgE and IL-4 in the AR group increased significantly compared with those in the Con group. After moxibustion treatment, both of them decreased significantly (**Figures 2B, C**).

### 3.3 Alpha diversity and beta diversity

**Figure 3:**
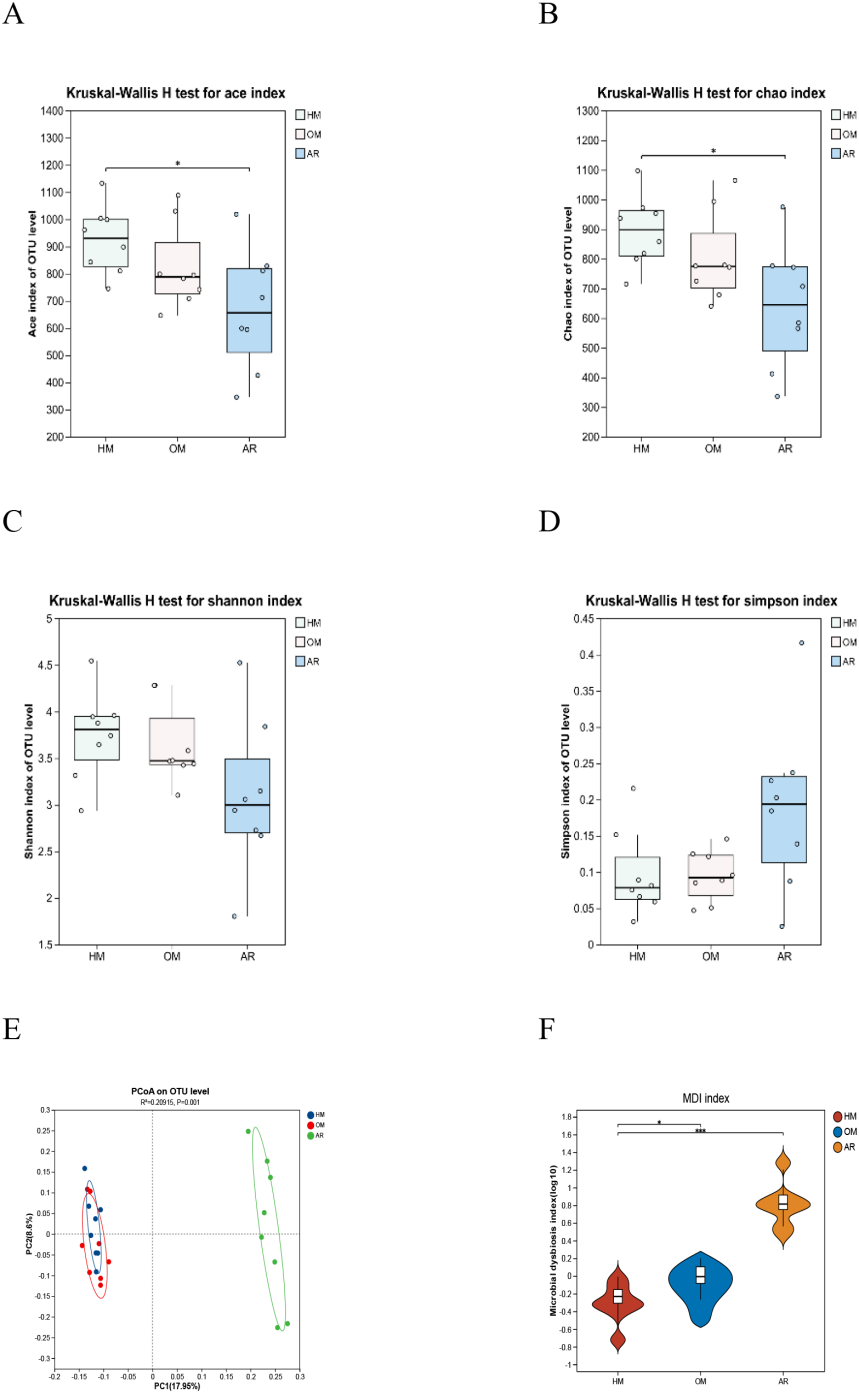
Heat-sensitive moxibustion (HM) altered the microbiota dysbiosis in ovalbumin-induced allergic rhinitis rats. **(A–D)** Alpha diversity analysis (Ace index, Chao index, Shannon index, and Simpson index). **(E)** Principal coordinates analysis (PCoA) (n = 8). **(F)** Microbial imbalance index (MDI). It is a definite index for assessing the degree of microbial ecological imbalance. The larger the value, the greater the degree of microbial flora disorder. *p < 0.05; ***p < 0.001.

To determine the taxonomic composition of the gut microbiota and differences in microbial diversity, alpha- and beta-diversity analyses were performed. Alpha diversity was used to analyze the richness (number of taxa) and evenness (abundance distribution of groups) within the same microbial community. Ace, Chao, Shannon, and Simpson indices were assessed for each sample. The Ace index, analyzed using the Wilcoxon test, showed a significant difference between the HM and AR groups and no difference between the OM and AR groups. The Chao index showed a significant difference between the NM and AR groups and no difference between the OM and AR groups. There was no difference in the Shannon index and Simpson index between the HM group and AR group (**Figures 3A–D**). Next, beta-diversity analysis was used to generate a weighted UniFrac PCoA and showed the clusters between the HM, OM, and AR groups. As shown in **Figure 3E**, the HM group and AR group showed different trends, compared with the OM group, which showed a similar trend.

#### 3.3.1 Flora imbalance index

The intestinal flora imbalance index [microbial imbalance index (MDI)] is an index that determines the degree of microbial imbalance. The higher the value, the greater the degree of bacterial disturbance. We observed that there were significant differences in intestinal flora between the HM group and AR group, and between the HM group and OM group. These results indicated that the intestinal disorder in the HM group was significantly lower than that in the AR group (p < 0.001), and the intestinal disorder in the HM group was also significantly lower than that in the OM group (p < 0.05) (**Figure 3F**).

**Figure 4:**
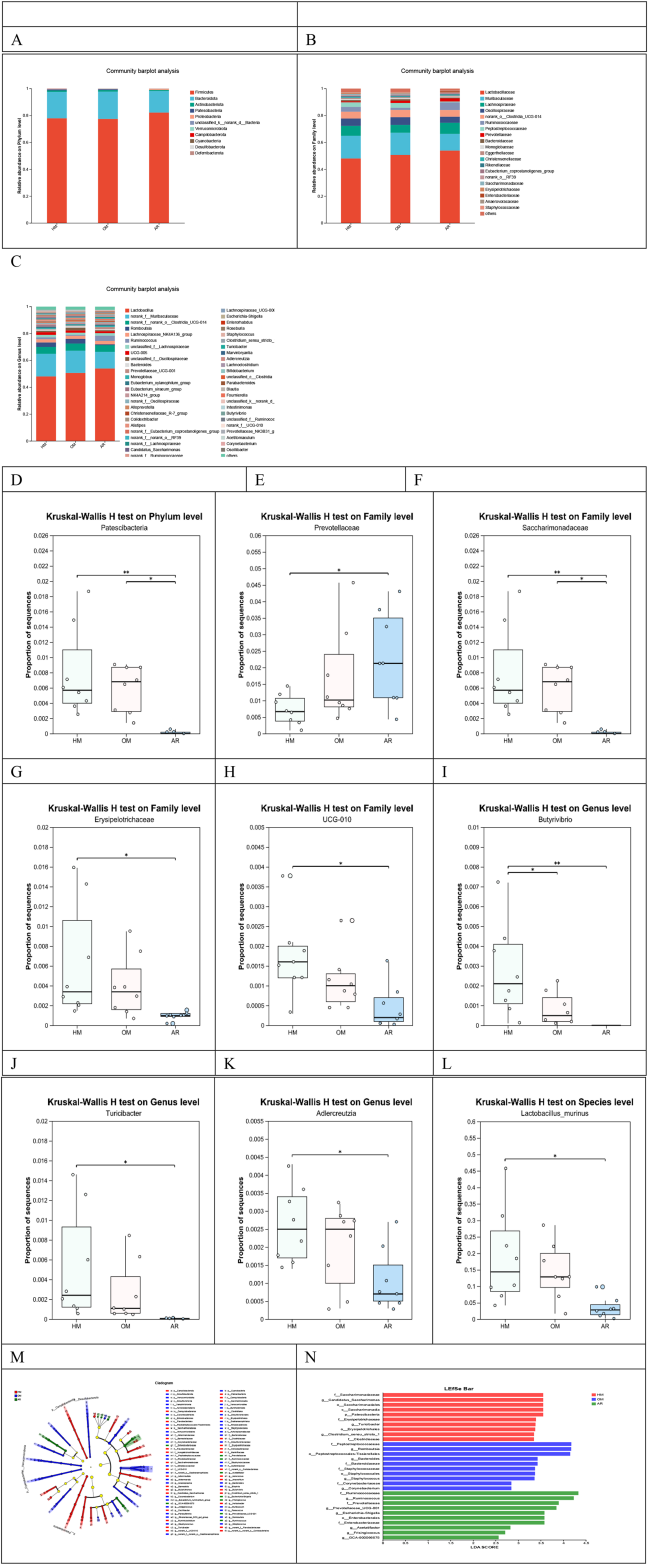
Heat-sensitive moxibustion (HM) altered the gut microbiota dysbiosis in ovalbumin-induced allergic rhinitis rats. The top 20 species with the highest horizontal abundance of phylum and family, and the top 50 species with the highest horizontal abundance of genus, are colored differently **(A–C)**, and the key genera, such as Patescibacteria **(D)**, Prevotellaceae **(E)**, Saccharimonadaceae **(F)**, Erysipelotrichaceae **(G)**, UCG-010 **(H)**, *Butyrivibrio* **(I)**, *Turicibacter* **(J)**, *Adlercreutzia* **(K)**, and *Lactobacillus murinus* **(L)**, are presented. Linear discriminant analysis (LDA) score map and cladogram (LDA fold = 3.5 and p < 0.05). **(M)** Cladogram. **(N)** LDA score map. The histogram’s length represents the LDA value’s size. The circles represent the phylum, class, order, family, and genus from the inside to the outside. Each small circle at different classification levels represents a classification at that level. The diameter of the small circle is proportional to the relative abundance. *p < 0.05; **p < 0.01.

In order to analyze the changes in microbiota diversity differences, considering the two groups as treatment groups, we used histograms to show the composition of the dominant microbiota in the three groups and observed significant changes in the microbiota at different levels. According to the species annotation results, the top 20 species with the highest horizontal abundance of family and phylum and the top 50 species with the highest horizontal abundance of genus were selected to generate relative abundance histograms so as to visualize the species with high relative abundance and their proportions at different taxonomic levels (**Figures 4A–C**).

At the phylum level, Firmicutes and Bacteroidetes are the most abundant microbiota. Compared with the AR group, Patescibacteria in the HM group were higher than those in the AR group (p < 0.01), while those in the HM group were higher than those in the AR group (p < 0.05) (**Figure 4D**). At the family level, compared with those in the AR group, Prevotellaceae were significantly decreased in the HM group (p < 0.05) (**Figure 4E**), and Saccharimonadaceae, Erysipelotrichaceae, and UCG-010 were significantly increased (p < 0.05 or p < 0.01). Prevotellaceae, Erysipelotrichaceae, and UCG-010 showed no statistically significant differences for the OM group (**Figures 4F–H**). At the genus level, compared to those in the AR group, *Butyrivibrio*, *Turicibacter*, *Adlercreutzia*, and *Lactobacillus murinus* were significantly increased in the HM group (p < 0.01 and p < 0.05), while there were no statistically significant differences for the OM group. The predominant bacteria at the genus level were *Lactobacillus* (**Figures 4I–L**). However, there were no statistically significant differences between the two groups. *Lactobacillus* showed an increased trend (47.99% vs. 50.61%) compared with the OM group. However, we found significant changes in *L. murinus* at the species level: the HM group was significantly higher than the AR group, and the OM group was not significantly different from the AR group. It is possible that both the HM group and the OM group function as treatment groups, resulting in a relatively minor difference in bacterial flora. To assess the differences between these two groups, we utilized the AR group as a comparative baseline.

In addition, *Lactobacillus*, Romboutsia, Lachnos piraceae_NK4A136_group, and UCG-005 dominated the microflora at the generic level. The values of *Candidatus*_Saccharimonas and *Butyrivibrio* in the HM and OM groups were higher than those in the AR group (p < 0.01), while those in the HM group were higher than those in the OM group (p < 0.05). The proportions of *Turicibacter* and *Adlercreutzia* in the HM group were higher than those in the AR group (p < 0.05), but there was no significant difference between the OH group and the AR group. The results showed that Patescibacteria (p < 0.01), Saccharimonadaceae (p < 0.01), Erysipelotrichaceae (p < 0.05), UCG-010 (p < 0.05), *Butyrivibrio* (p < 0.01), *Turicibacter* (p < 0.05), *Adlercreutzia* (p < 0.05), and *L. murinus* (p < 0.05) were significantly decreased in the HM group, whereas Prevotellaceae (p < 0.05) was significantly increased compared with that in the AR group.

LEfSe analysis is an LDA of a sample based on different grouping conditions and taxonomic composition to identify communities or species that express significant differences in the sample division. LEfSe analysis was used to explore the intestinal flora with significant statistical significance and biological correlation between the HM and OM groups, as represented by LDA score plot (**Figure 4N**) and branching plot (**Figure 4M**), with LDA multiple = 3.5, p < 0.05. Several microbiota, such as f:Prevotellaceae and f:Prevotellaceae_UCG-001, were significantly increased in the AR group. Several microbiota, such as f:Peptostreptococcaceae and g:Romboutsia:Peptostreptococcales-Tissierellales, were significantly enriched in the OM group. In the HM group, f:Saccharimonadaceae, o:Saccharimonadales, c:Saccharimonadia, p:Patescibacteria, and other microorganisms were enriched. The high-throughput sequencing results indicate that HM significantly increased the relative abundance of species such as Patescibacteria, Saccharimonadaceae, UCG-010, *Butyrivibrio*, *Turicibacter*, *L. murinus*, and *Adlercreutzia* while decreasing the relative abundance of Prevotellaceae. This shift in microbial composition is conducive to improving the gut microbiota of AR rats.

### 3.4 Metabolomics analysis of heat-sensitive moxibustion in AR rats

**Figure 5:**
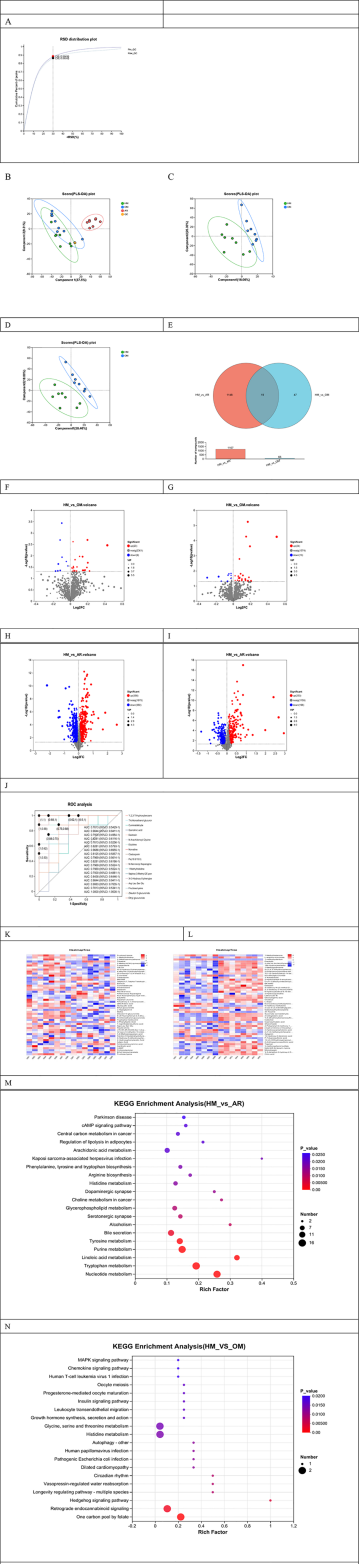
Characteristics of metabolomics in urine. Relative standard deviation (RSD) distribution plot. **(A)** Principal component analysis (PCA) of the three groups of urine metabolomics. **(B)** Principal component analysis (PCA) of the HM group and the OM group in the positive ions **(C)** and negative ions **(D)**. Dots of the same color represent each biological repetition in the group, and the distribution state of the dots reflects the differences between and within the groups. The Venn diagram **(E)** shows the number of common and specific metabolites in different groups. **(F–I)** Volcano plots. **(J)** receiver operating characteristic (ROC). The hierarchical clustering analysis of HM vs. allergic rhinitis (AR) **(K)** and HM vs. OM **(L)**. KEGG enrichment analysis of metabolites significantly regulated by HM vs. AR **(M)** and HM vs. OM **(N)**.

Considering the different composition of the microbiota between the HM and OM groups, to further identify the unique potential mechanism of heat-sensitive moxibustion for AR, we performed untargeted metabolomics determination of the three groups using UHPLC-Q Exactive HF-X. The RSD distribution plot demonstrates that the analysis process has excellent reproducibility and that the data quality is reliable (**Figure 5A**).

PLS-DA is a supervised discriminant analysis method. It was found that the PLS-DA plot showed a clear separation of the HM sample from the AR sample, with a slight trend of dissociation from the AR group (**Figure 5B**). To further screen out the significantly different metabolites between the two comparison groups, two-group comparisons between the HM and OM groups were also performed. In the positive ion mode, the PLS-DA plot showed a clear separation of the HM samples from the OM samples, a trend also reflected in the negative ion mode. The goodness of fit was satisfactory (**Figures 5C, D**). In the positive ion mode, a total of 32 differential metabolites were identified in the HM and OM groups, with 23 metabolites upregulated and nine metabolites downregulated (**Figure 5F**). A total of 718 differential metabolites were identified in the HM and OM groups, with 358 metabolites upregulated and 360 downregulated (**Figure 5H**); 34 were identified between the HM and OM groups, including 24 upregulated and 10 downregulated (**Figure 5G**). A total of 449 differential metabolites were identified between the HM and AR groups, with 253 upregulated and 196 downregulated (**Figure 5I**). To visually demonstrate common or unique metabolites among individual metabolic sets, the results are shown using Venn diagram analysis: there were 19 potential biomarkers between the HM group and AR group, and between the HM group and OM group, which play potentially important roles in the heat-sensitive moxibustion group, common moxibustion group, and model group (**Figure 5E**). These potential biomarkers were then used to generate the predictive receiver operating characteristic (ROC) curves (**Figure 5J**). All area under the curve (AUC) values of the biomarkers were greater than 0.70, indicating that these 19 metabolites could be considered as potential markers.

The results of hierarchical clustering analysis are shown in **Figures 5K, L**; by clustering the differentially expressed metabolites, we can intuitively see the change trend of different metabolites in different groups. The metabolites clustered in the same cluster have similar expression patterns, may have similar functions, or participate in the same metabolic processes or cellular pathways. The color in the figure indicates the relative expression of metabolites in this group. Compared with the AR group, the HM group had significantly changed metabolites, including citric acid and 16β-hydroxyestradiol; compared with the OM group, the HM group also had changed metabolites, including fructosyl-lysine, l-hypoglycin A, and cAMP.

We performed KEGG pathway enrichment analysis for differential metabolites. The results showed that the metabolites of MH and OM were mainly involved in the MAPK signaling pathway, glycine, serine, and threonine metabolism, histidine metabolism, etc. (**Figure 5N**). The metabolites of MH and AR were mainly involved in nucleotide metabolism, tryptophan metabolism, and linoleic acid metabolism. After screening, we found that there were seven intersection pathways of HM vs. AR and HM vs. OM: glutamatergic synapse, dopaminergic synapse, Kaposi sarcoma-associated herpesvirus infection, cocaine addiction, melanogenesis, alcoholism, and histidine metabolism (**Figure 5M**).

**Figure 6:**
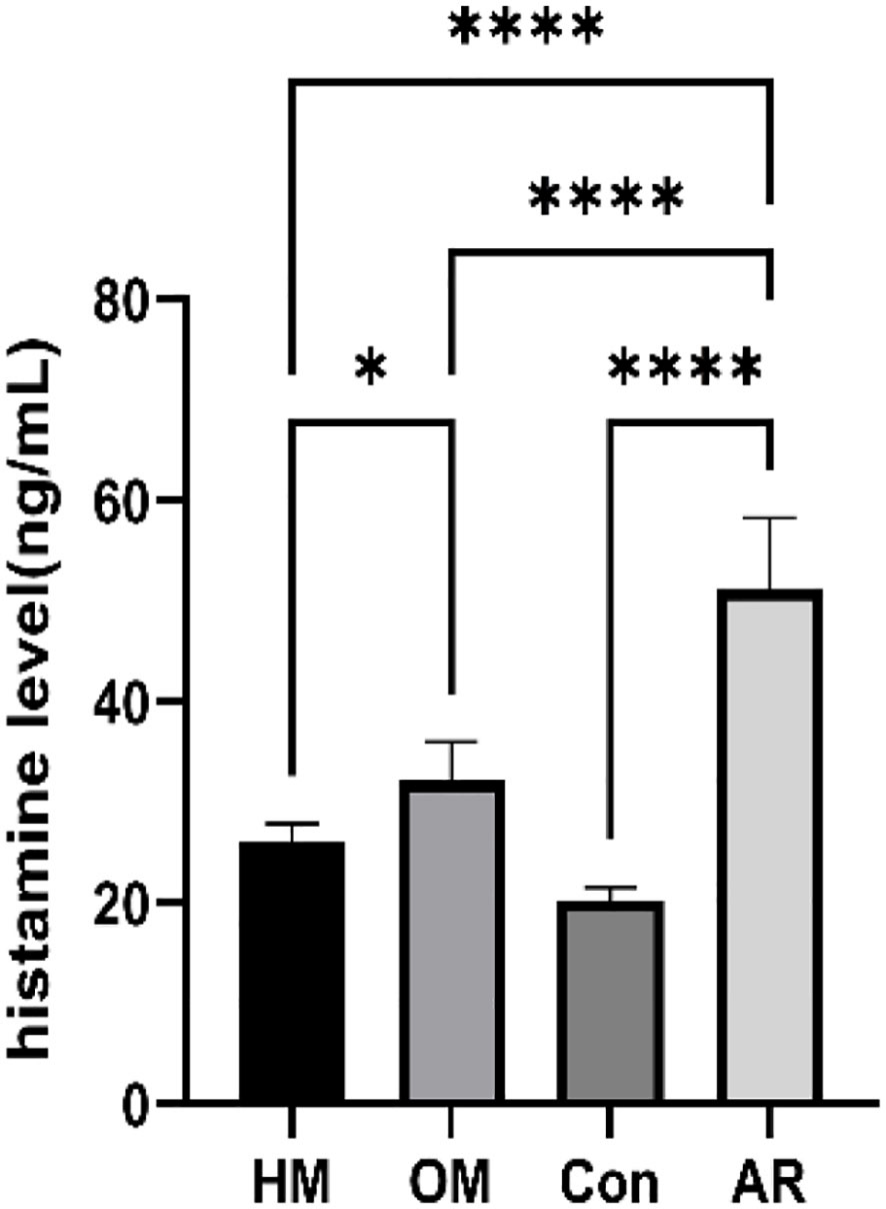
Nasal mucosa histamine ELISA (n = 8). *p < 0.05; ****p < 0.0001.

This finding suggests that the histidine metabolism pathway is a core mechanism through which moxibustion exerts its anti-allergic effects and that heat-sensitive moxibustion (HM) and conventional moxibustion (OM) may differentially regulate this pathway. To clarify the activity changes in this pathway, we measured the levels of histamine—a key downstream effector molecule—using ELISA. As shown in **Figure 6**, histamine concentration in the nasal mucosa was significantly elevated in the AR model group (AR) compared to the blank control group (Con) (p < 0.0001). Following intervention with either HM or OM, histamine levels decreased markedly compared to those in the AR group (p < 0.0001). Compared with OM, the histamine concentration in HM was significantly decreased (p < 0.05).

### 3.5 Correlation between metabolomics characteristics and microbial communities

**Figure 7:**
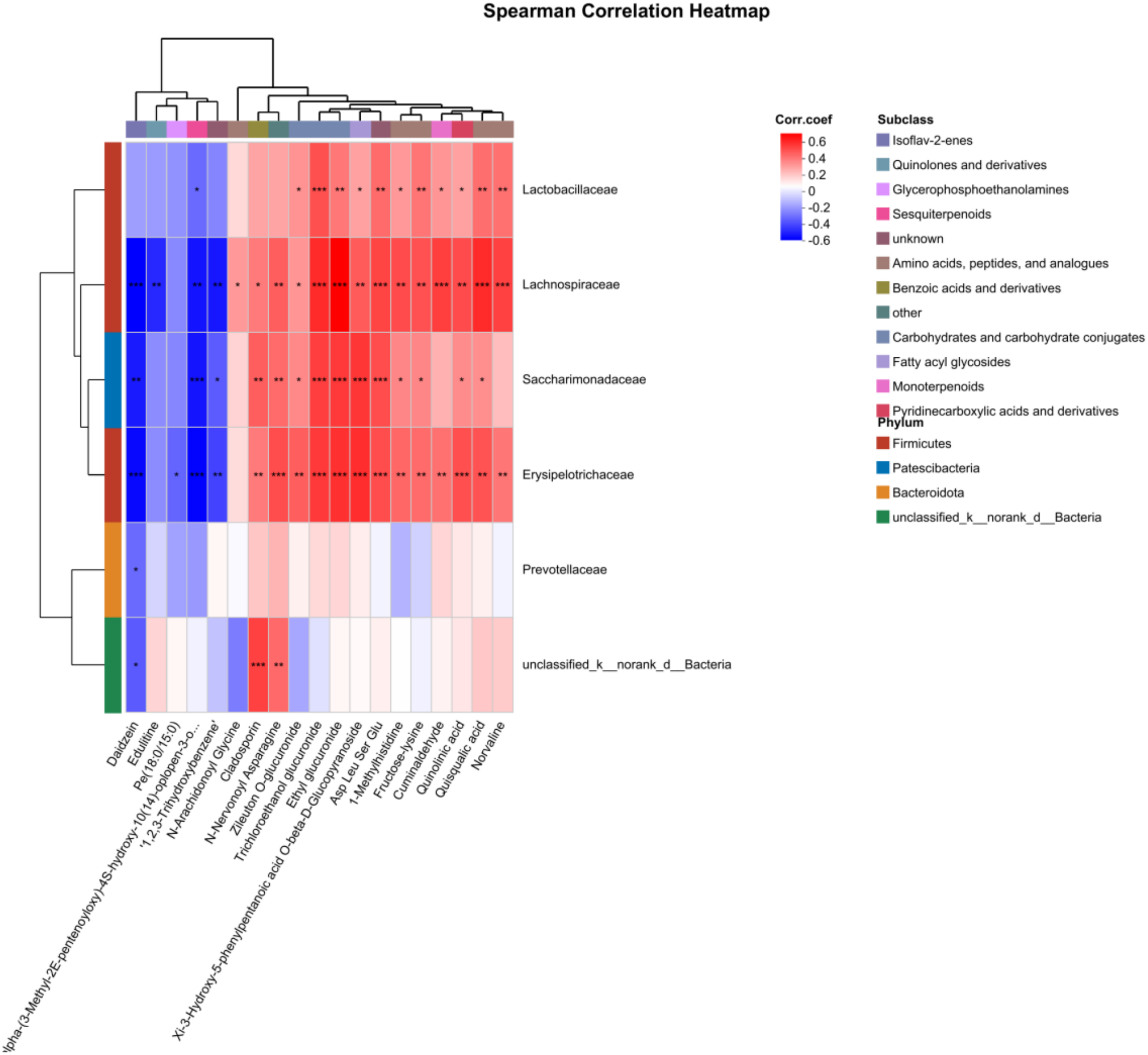
Correlation analysis of urine metabolites and gut microbiota. *p < 0.05; **p < 0.01, ***p < 0.001.

In order to study the correlation between changes in metabolite levels and microbiome composition, Spearman’s correlations were performed on the differentiated species sets and differentiated metabolic sets screened from the microbiome (**Figure 7**). Multiple correlation analysis showed that Erysipelotrichaceae, Saccharimonadaceae, and Lachnospiraceae were positively correlated with norvaline, quisqualic acid, Asp Leu Ser Glu, and other metabolites. They were negatively correlated with daidzein, 9alpha-(3-methyl-2*E*-pentenoyloxy)-4*S*-hydroxy-10(14)-oplopen-3-one and “1,2,3-trihydroxybenzene”. In addition, Lactobacillaceae were positively correlated with ethyl glucuronide, zileuton *O*-glucuronide, trichloroethanol glucuronide, and other metabolites. They were negatively correlated with 9alpha-(3-methyl-2*E*-pentenoyloxy)-4*S*-hydroxy-10(14)-oplopen-3-one; Prevotellaceae were negatively correlated with daidzein. These results show that there is a close correlation between the nine microorganisms selected and the 19 metabolites selected. Heat-sensitive moxibustion can regulate the disturbance of microorganisms and metabolites in AR treatment.

## 4 Discussion

According to the current AR treatment, there are still problems such as low long-term treatment compliance, large side effects, and high economic burden for patients. Therefore, it is necessary to explore new therapeutic methods from different angles. In this study, in order to find the markers under the effect of heat-sensitive moxibustion, a standardized AR rat model was established. The successful OVA modeling was judged according to behavioral scores, and then 16S ribosomal DNA (rDNA) sequencing and metabolomics analysis were performed on the feces and urine of the successfully modeled rats.

Regarding how the thermal stimulation of the distant acupoint BL13 affects the composition of the intestinal microbiota, we suspect that the most reasonable mechanism involves the neuro-immune-endocrine network mediated by the gut–brain axis. Specifically, heat-sensitive moxibustion at BL13 may activate local sensory nerves, with afferent signaling converging on the central nervous system to enhance vagal tone (23). Vagal activation subsequently modulates gut function through the cholinergic anti-inflammatory pathway, effectively suppressing intestinal inflammation and shaping the immune microenvironment (24). This integrated neuro-immune-gut axis framework offers a biologically coherent explanation for the distant regulatory effects of acupoint stimulation observed in our study.

In previous reports, Patescibacteria were negatively associated with OVA (25), Prevotellaceae were significantly increased in AR diseases, and Arrieta et al. reported that intestinal Prevotellaceae family induced TH2 immune response (26). In TH1/TH2 disorders, the imbalance of Th2 dominance is closely related to the incidence of AR (27). According to the findings made in a previous study (12), an elevated abundance of *Prevotella* was found in the respiratory microbiome of patients with allergic rhinitis. The increase in *Prevotella* in the upper respiratory tract of AR patients may be related to the enlargement of the inferior turbinates during the allergic state, which increases epithelial permeability. This allows for abnormal fluid accumulation on the airway surface and affects the balance of the commensal microbiota in the nasal mucosa. In this paper, the level of Patescibacteria in the HM group was higher than that in the AR group (p > 0.05). There was no significant difference between the OM group and the AR group, and the negative correlation with OVA was consistent with previous studies. Compared with OM, HM has the ability to increase the intestinal Patescibacteria in mice, which may be the therapeutic target of HM, different from ordinary moxibustion (28). *Prevotella* is the most representative genus in the Prevotellaceae family. It was discovered that a reduced abundance of oral *Prevotella* in subjects with peanut allergy was significantly correlated with lower oral short-chain fatty acid (SCFA) levels. Furthermore, oral administration of *Prevotella* decreased the local secretion of Th2-stimulating epithelial factors, such as IL-33 and TSLP, as well as Th2 cytokines, including IL-4, IL-5, and IL-13. This is contrary to the previous report, and the reason may be related to the inconsistency of the detection site.

Changes in the abundance of UCG-010 are associated with the occurrence and development of the disease. The reduction of UCG-010 in the gut affects the balance and metabolic function of the gut flora (29). A recent study showed that the abundance of UCG-010 was positively correlated with the levels of SCFAs in the gut (30). In addition, Meixia Chen et al. found that UCG-010 may play an important role in anti-inflammation (31). Our study showed that UCG-010 decreased in the model group, while the changes were reversed in the HM group (p < 0.05), and there was no significant difference in the ordinary moxibustion group. Compared with ordinary moxibustion, we speculated that heat-sensitive moxibustion may achieve a therapeutic effect through the elevation of UCG-010. SCFAs are also produced by the genus *Butyrivibrio* (32).

SCFAs affect the activation and effector function of T cells and are more resistant to allergic processes in the lungs (33). In particular, they affect the polarization and activation of Th1, Th2, and Th17, and studies have shown that SCFAs produced in the gut can impair Th2 polarization (34). *Turicibacter* is a genus of healthy bacteria in the gut microbiome, which is considered to have anti-inflammatory effects (35). *Turicibacter* can affect the production of some mediators, thereby reducing the inflammatory response (36). Consistent with previous experiments (37), in this paper, the *Turicibacter* in the HM group was the highest, and there was a significant difference between the HM group and the AR group (p > 0.05), while there was no significant difference between the AR group and the ordinary moxibustion group. We concluded that the HM group may have a therapeutic effect by modulating *Turicibacter* compared to the OM group.

The association of *Adlercreutzia* with allergic rhinitis has never been previously reported, and the role of *Adlercreutzia* in AR has not been explored; however, its presence in the important results of our analysis suggests that it may be a target of future interest. In some intestinal inflammatory diseases, such as inflammatory bowel disease, the abundance of *Adlercreutzia* is reduced, and this imbalance may lead to a disturbance of the gut microbial ecosystem (38). In this paper, the AR group was lower than the HM group (p < 0.05), indicating that the heat-sensitive moxibustion group reversed intestinal disorders, while the ordinary moxibustion group had no significant difference (39). Our research shows that *L. murinus* may be a key factor in the development of AR. It could be used as a diagnostic biomarker for AR and was negatively correlated with IgE, with a significant difference between the HM and AR groups (p < 0.05), and no significant difference between the OM and AR groups. The evidence suggests that *L. murinus* may be a new target for treating AR that is different from OM. Research has confirmed that *L. murinus* directly targets polyamine metabolic enzymes in host intestinal cells by secreting small RNA molecules (such as sR-182871, sR-242825, and sR-257800), inhibits polyamine synthesis, and thereby regulates the host’s immune status (40). Some articles have shown that patients with AR have significantly lower abundance of *L. murinus* in the nasal cavity compared to healthy controls and patients with non-allergic rhinitis, and the abundance of this bacterium is significantly negatively correlated with serum IgE levels (39). This suggests that the abnormal abundance of *L. murinus* may be a cross-site indicator of the systemic microbiota–immune imbalance in AR. This study further confirmed in the AR rat model that after intervention with heat-sensitive moxibustion, the abundance of *L. murinus* in the intestine significantly increased, and it showed a synchronous negative change with serum IgE levels, forming a “animal–human” cross-species correspondence. This confirmed that the association between *L. murinus* and AR is not accidental but a common rule regulated by the intestinal–metabolism axis in controlling AR.

Metabolomics is a comprehensive characterization of metabolites in biological systems that can generate unique chemical fingerprints for specific cellular processes. In particular, metabolomics is increasingly being used to diagnose diseases, understand disease mechanisms, identify new drug targets, and monitor treatment outcomes. Metabolomics may provide unique and novel insights. In this study, heat-sensitive moxibustion induced 19 metabolic profiles in rats—1-methylhistidine, xi-3-hydroxy-5-phenylpentanoic acid *O*-beta-d-glucopyranoside, cladosporin, cuminaldehyde, daidzein, Pe(18:0/15:0), *N*-nervonoyl asparagine, edulitine, *N*-arachidonoyl glycine, 9alpha-(3-methyl-2*E*-pentenoyloxy)-4*S*-hydroxy-10(14)-oplopen-3-one, quisqualic acid, ethyl glucuronide, zileuton *O*-glucuronide, trichloroethanol glucuronide, Asp Leu Ser Glu, quinolinic acid, and norvaline. There were significant changes in “1,2,3-trihydroxybenzene” and fructose-lysine. These metabolites may be potential markers of heat-sensitive moxibustion in the treatment of allergic rhinitis. Our metabolomics analysis revealed a significant increase in cuminaldehyde levels in rats treated with heat-sensitive moxibustion (HM) compared to the AR model group. However, key evidence from a study on *Atractylodes macrocephala* volatile oil demonstrates that cuminaldehyde can be explicitly generated as a functional metabolite through gut microbiota remodeling (41). This provides a plausible mechanistic explanation for our observation: the increase in cuminaldehyde is likely a downstream consequence of HM-induced restructuring of gut microbial metabolism. Given its documented multifactorial anti-inflammatory activity, including interactions with COX-2, which is activated in the AR state (42), cuminaldehyde may contribute to the systemic anti-inflammatory effects of HM (43, 44).

Regarding 1-methylhistidine, we speculate that *Lactobacillus* may enhance the integrity of the intestinal epithelial mechanical barrier through its ability to produce butyrate and regulate intestinal barrier function (45). This process not only secures the supply of precursors for the endogenous synthesis of 1-methylhistidine but also ensures that anserine (the exogenous precursor of 1-methylhistidine) is fully hydrolyzed by host peptidases in the small intestine. Consequently, it minimizes the risk of anserine entering the large intestine and being excessively degraded by the gut microbiota, thereby maintaining the metabolic balance of 1-methylhistidine (46).

It is critical to note that our findings demonstrate an association, not causation. The present study design cannot establish whether gut microbiota alterations cause the therapeutic effects of HM on AR. Although specific changes in metabolites (cuminaldehyde and 1-methylhistidine) and microbes (e.g., *L. murinus* and Patescibacteria) are linked to symptom relief, they may be secondary to other primary effects of Heat-sensitive moxibustion (HSM), such as systemic anti-inflammation. To establish causality, future work should employ fecal microbiota transplantation (FMT) into germ-free or antibiotic-treated AR models. A successful transfer of the HM phenotype would strongly support a causal role. Conversely, ablating the gut microbiome with antibiotics prior to HM will test its necessity for treatment efficacy. Such interventional studies are pivotal to conclusively validate the gut–metabolite axis as a direct target of HM.

Our multi-omics analysis first identified a compelling association between heat-sensitive moxibustion (HM) and the histidine metabolism pathway. This was a pivotal discovery, as this pathway is responsible for the biosynthesis of histamine—a central mediator in AR (47). Histidine serves as the direct precursor to histamine, a conversion catalyzed by histidine decarboxylase. Upon its release, histamine activates receptors such as H1R and H4R on immune cells, triggering the release of pro-inflammatory cytokines (e.g., IL-6, TNF-α, and IL-8) and driving the hallmark symptoms of AR (48, 49). Therefore, modulating this pathway could directly impact the allergic response. To functionally validate this metabolomics prediction, we directly measured the pathway’s key effector and found that HM treatment significantly reduced histamine levels in the nasal mucosa compared to the AR model group. HM demonstrated a significant trend toward superior efficacy in reducing tissue histamine compared to OM. This observation aligns with our initial KEGG analysis, and its effect may be related to a more efficient regulation of the histidine metabolic pathway.

In addition to neural mechanisms, the unique delivery route of moxibustion—specifically, the inhalation of bioactive smoke volatiles—deserves careful consideration. Emerging evidence indicates that environmental flavonoids, particularly those originating from indoor sources, may confer protection against asthma and allergic rhinitis (50). Given the high concentration of flavonoids in mugwort (*Artemisia argyi*), we propose that volatile flavonoids and aromatic compounds released during moxibustion combustion can be inhaled, potentially contributing to the anti-allergic effects of HM. These inhaled compounds may exert local anti-inflammatory and immunomodulatory actions in the respiratory tract, possibly complementing systemic effects mediated via the gut–brain axis to alleviate AR. Although the inhalation pathway was not directly evaluated in this study, it represents a plausible mechanism warranting further exploration. Future research should characterize the volatile organic compound profile of HM smoke and assess the therapeutic potential of its key constituents in experimental models of allergic airway inflammation.

## 5 Conclusion

This study demonstrates that heat-sensitive moxibustion alleviates allergic rhinitis through a multi-targeted mechanism involving both the modulation of specific gut microbiota (notably *L. murinus*, Patescibacteria, *Butyrivibrio*, and *Turicibacter*)—which is closely associated with alterations in key metabolites (cuminaldehyde and 1-methylhistidine)—and the regulation of histidine metabolism. The unique “heat-sensitization” effect further enhances its superior therapeutic efficacy.

To our knowledge, this represents the first investigation to establish comprehensive correlations between gut microbiota and urinary metabolomics profiles in an AR model. Our findings confirm the therapeutic role of heat-sensitive moxibustion in AR recovery and provide mechanistic insights supporting its clinical application, thereby proposing a novel strategic approach for AR treatment.

## Data Availability

The datasets presented in this study can be found in online repositories. The names of the repository/repositories and accession number(s) can be found below: PRJNA1286800 (SRA).
